# nhmmer: DNA homology search with profile HMMs

**DOI:** 10.1093/bioinformatics/btt403

**Published:** 2013-07-09

**Authors:** Travis J. Wheeler, Sean R. Eddy

**Affiliations:** HHMI Janelia Farm Research Campus, Ashburn, VA 20147, USA

## Abstract

**Summary:** Sequence database searches are an essential part of molecular biology, providing information about the function and evolutionary history of proteins, RNA molecules and DNA sequence elements. We present a tool for DNA/DNA sequence comparison that is built on the HMMER framework, which applies probabilistic inference methods based on hidden Markov models to the problem of homology search. This tool, called nhmmer, enables improved detection of remote DNA homologs, and has been used in combination with Dfam and RepeatMasker to improve annotation of transposable elements in the human genome.

**Availability:** nhmmer is a part of the new HMMER3.1 release. Source code and documentation can be downloaded from http://hmmer.org. HMMER3.1 is freely licensed under the GNU GPLv3 and should be portable to any POSIX-compliant operating system, including Linux and Mac OS/X.

**Contact:** wheelert@janelia.hhmi.org

## 1 INTRODUCTION

A widely used general purpose tool for DNA/DNA sequence comparison is blastn (Altschul *et al.*, 1990; Camacho *et al.*, 2009), which heuristically approximates the Smith–Waterman algorithm (Smith and Waterman, 1981) for recognizing local regions of similarity between two sequences. In recent years, most advances in DNA/DNA comparison have related to accelerating search for near-exact matches (Kent, 2002; Langmead *et al.*, 2009; Li and Durbin, 2009), and to improving whole-genome alignment (Kurtz *et al.*, 2004; Schwartz *et al.*, 2003). Another area that deserves attention is the development of methods that maximize the power of computational sequence comparison tools to detect remote homologies.

Profile hidden Markov models (profile HMMs) (Durbin *et al.*, 1998; Krogh *et al.*, 1994) represent an important advance in terms of sensitivity of sequence searches for remote homology. They provide a formal probabilistic framework for sequence comparison and improve detection of remote homologs by (i) enabling position-specific residue and gap scoring based on a query profile, and (ii) calculating the signal of homology based on the more powerful ‘Forward/Backward’ HMM algorithm that computes not just one best-scoring alignment, but a sum of support over all possible alignments. In the past, this improved sensitivity came at a significant computational cost, but recent advances in HMMER3 have increased speed for protein search by ∼100-fold, reaching blastp-like speed through a combination of filtering heuristics (Eddy, 2008) and computer engineering (Eddy, 2011; Farrar, 2007). Tools based on profile HMMs (Eddy, 2009; Karplus *et al.*, 1998) have historically focused on protein search, with little concentration on the challenges presented by (i) chromosome-length target sequences, and (ii) the extreme composition bias often seen in genomic DNA. With attention to the details of DNA search, nhmmer builds upon the speed advances of HMMER3, bringing the power of profile HMMs to DNA homology search, at speeds nearly as fast as blastn with sensitive settings.

An example of a biological problem requiring sensitive detection of remote DNA homologs is the annotation of genomic sequence derived from ancient transposable element (TE) expansions. A prerelease version of the nhmmer tools has recently been shown to provide increased sensitivity over blastn and other single-sequence search methods, with reduced false discovery rate and reasonable runtime, in searching for TEs (Wheeler *et al.*, 2013). For example, when nhmmer was used within the recently released RepeatMasker 4.0 (Smit and Hubley, 2013), an additional 150 Mb (5%) of the human genome was reliably annotated as derived from TEs.

## 2 USAGE AND PERFORMANCE

**Usage.** The program nhmmer is used to search one or more nucleotide queries against a nucleotide sequence database. For each query, nhmmer searches the target database and outputs a ranked list of the hits with the most significant matches to the query. A query may consist of a single sequence, a multiple sequence alignment, or a profile HMM built using the HMMER program hmmbuild. Each hit represents a region of local similarity between a portion of the query and a subsequence of the full target database sequence, and is assigned a similarity score S in bits, along with an *E*-value (Eddy, 2008) indicating the expected number of false positives at a threshold of score S. Each hit is also accompanied by an alignment of the matched sequence to the model, with values indicating the confidence with which each position is aligned.

The final score, boundaries and alignment of a hit are computed based on filling in a Forward/Backward dynamic programming matrix, but the computational burden of doing this for the full target database is prohibitive. Therefore, nhmmer uses a series of acceleration filters that depend on simpler approximations of the final Forward score of a hit. These filters are based on those used in the HMMER3 protein search tools (Eddy, 2011), but have been modified to work in the context of long (potentially chromosome length) target sequences. The initial filter, called ‘single segment ungapped Viterbi’, scans along the target sequence with a fast ungapped Viterbi alignment using a reduced-precision, 16-way vector-parallel approach (Farrar, 2007). Windows around high-scoring ungapped alignments are subjected to a full-gapped Viterbi alignment to the model. Candidate alignments passing this filter then endure the full rigor of a Forward/Backward alignment to the query, including application of a context-dependent null model to account for composition bias shared by the query and target. For more details on the full acceleration pipeline, see Eddy and Wheeler (2013).

**Fig. 1.**
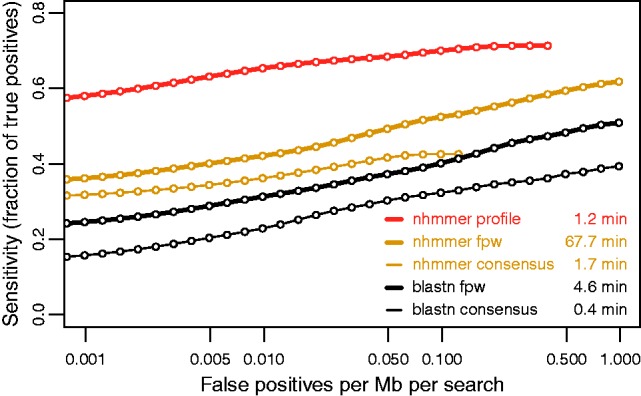
Benchmark of search sensitivity and specificity. Searches were performed against the Rmark3 benchmark either by constructing a single profile HMM from the query alignment (nhmmer profile), constructing a consensus sequence from the query alignment (consensus), or by using family pairwise search (*fpw*). The aborted lines for two nhmmer variants indicate that the list of all hits found by each search variant was exhausted before reaching 1 false positive per Mb per search. The nhmmer parameters were default, except setting the *E*-value threshold, ‘-E 100’ for profile and consensus variants, to extend the hit list. Higher *E*-values have no effect, as further hits (true and false) are filtered by the default acceleration heuristics. Many parameters were tested for NCBI blastn 2.2.28+, with the best-performing variant shown here (‘-word_size 7 -penalty -3 -reward 2 -gapopen 4 -gapextend 2’). For each combination of program and method, hits for all families were collected and ranked by *E*-value, and true and false hits were defined as described in the text. The *Y*-axis is the fraction of 780 true positives detected with an *E*-value sufficient to achieve the false-positive rate specified on the *X*-axis. Runtime was collected on a single thread on a 2.66 GHz Intel Gainestown (X5550) processor. The benchmark can be downloaded from http://selab.janelia.org/publications.html

**Performance.** In Figure 1 we consider the performance of nhmmer on a benchmark called Rmark3 that has been used previously to test the RNA homology search tool Infernal (Nawrocki *et al.*, 2009). The benchmark consists of 106 families from Rfam that could be divided into two groups such that no sequence in one group is >60% identical to any sequence in the other group [Rfam 10.0, Gardner *et al*. (2011)]. One group was used as the query alignment for the family, and sequences from the other group (780 sequences in total) were embedded in 10 Mb of sequence simulated using a 15-state HMM trained on genomic sequence from a variety of organisms. A positive was defined as an embedded sequence with >50% length covered by a query from the same family; a negative was defined as any hit that mostly covers simulated sequence. For more details on construction of the benchmark, see Nawrocki and Eddy (2007).

In this benchmark, we begin with an alignment of multiple members of a DNA sequence family and aim to find more instances of the family in the target sequence database. The standard methods for this homology search problem (e.g. using blastn) involve searching the target database with a single query sequence, either (i) producing a consensus sequence to represent the sequence family, then using the consensus as query to search the database, or (ii) using the family pairwise (*fpw*) search method, in which each individual sequence from the family alignment is used as a query, the hit lists are merged, and overlapping hits are adjudicated by recording the hit with the best *E*-value (Grundy, 1998). Using both of these single-sequence query approaches on Rmark3, nhmmer achieves better sensitivity than blastn.

These single-sequence query methods do not, however, take full advantage of the information contained within the query alignment. In nhmmer, a profile HMM is built from the alignment, and represents the residue and indel distributions for each position, modeling the conservation patterns of the family in a way that is not possible with single-sequence queries. The benefits of profile search are two-fold: (i) search power is much greater than even with *fpw*, and (ii) search speed is roughly equivalent to that of searching with a single consensus sequence, as only one search is performed for the entire family, rather than one for each sequence in the query alignment as in *fpw*.

In addition to being more sensitive than blastn, nhmmer represents a nearly 100-fold increase in speed over previous implementations of DNA homology search with profile HMMs. For example, using the seed alignment for Dfam entry DF0000789 (a 338 position-long DNA transposon) to search against the human genome with a single thread took nhmmer 12 min to complete, whereas HMMER 1.8.5 completed in 782 min and SAM 3.5 (Hughey and Krogh, 1995; Karplus *et al.*, 1998) required 844 min.

**Other applications.** HMMER3.1’s nhmmer has recently been adopted as a search engine within the TE annotation tool, RepeatMasker 4.0 (Smit and Hubley, 2013), where in conjunction with Dfam, it supports a substantial boost in sensitivity in human DNA repeat annotation with better speed than the previous most sensitive method (Wheeler *et al.*, 2013). The core pipeline of nhmmer has also been incorporated as an acceleration filter for the RNA homology search tool Infernal, where it supports fast filtering with negligible loss in Infernal sensitivity (E.Nawrocki and S.R.Eddy, unpublished data). We anticipate that nhmmer will similarly benefit other domains of DNA sequence comparison that depend on discriminative detection of remote homologs.

## References

[btt403-B1] Altschul SF (1990). Basic local alignment search tool. J. Mol. Biol..

[btt403-B2] Camacho C (2009). BLAST+: architecture and applications. BMC Bioinformatics.

[btt403-B3] Durbin R (1998). Biological Sequence Analysis: Probabilistic Models of Proteins and Nucleic Acids.

[btt403-B4] Eddy SR (2008). A probabilistic model of local sequence alignment that simplifies statistical significance estimation. PLoS Comput. Biol..

[btt403-B5] Eddy SR (2009). A new generation of homology search tools based on probabilistic inference. Genome Inform..

[btt403-B6] Eddy SR (2011). Accelerated profile HMM searches. PLoS Comput. Biol..

[btt403-B7] Eddy SR, Wheeler TJ (2013). The HMMER3.1 user’s guide.

[btt403-B8] Farrar M (2007). Striped Smith-Waterman speeds database searches six times over other SIMD implementations. Bioinformatics.

[btt403-B9] Gardner PP (2011). Rfam: Wikipedia, clans and the “decimal” release. Nucleic Acids Res..

[btt403-B10] Grundy WN (1998).

[btt403-B11] Hughey R, Krogh A (1995). SAM: sequence alignment and modeling software system. Technical report *UCSC-CRL-95-07*.

[btt403-B12] Karplus K (1998). Hidden Markov models for detecting remote protein homologies. Bioinformatics.

[btt403-B13] Kent WJ (2002). BLAT–the BLAST-like alignment tool. Genome Res..

[btt403-B14] Krogh A (1994). Hidden Markov models in computational biology: applications to protein modeling. J. Mol. Biol..

[btt403-B15] Kurtz S (2004). Versatile and open software for comparing large genomes. Genome Biol..

[btt403-B16] Langmead B (2009). Ultrafast and memory-efficient alignment of short DNA sequences to the human genome. Genome Biol..

[btt403-B17] Li H, Durbin R (2009). Fast and accurate short read alignment with Burrows–Wheeler transform. Bioinformatics.

[btt403-B18] Nawrocki EP, Eddy SR (2007). Query-dependent banding (QDB) for faster RNA similarity searches. PLoS Comput. Biol..

[btt403-B19] Nawrocki EP (2009). Infernal 1.0: Inference of RNA alignments. Bioinformatics.

[btt403-B20] Schwartz S (2003). Human–mouse alignments with BLASTZ. Genome Res..

[btt403-B21] Smit AFA, Hubley R (2013). RepeatMasker.

[btt403-B22] Smith TF, Waterman MS (1981). Identification of common molecular subsequences. J. Mol. Biol..

[btt403-B23] Wheeler TJ (2013). Dfam: a database of repetitive DNA based on profile hidden Markov models. Nucleic Acids Res..

